# Supplementary material for: Chromothripsis during telomere crisis is independent of NHEJ, and consistent with a replicative origin
Source: Genome Res. 2019 May;29(5):737–49. doi: 10.1101/gr.240705.118 (PMC6499312; doi:10.1101/gr.240705.118)
Supplement: Supplemental Material [file supp_gr.240705.118_Supplemental_file_1.zip › contigs/annotated_contigs/DB73/contig.3.DB73_length_599_mean_cov_9.39398998331.docx]

**DB73_length_599_mean_cov_9.39398998331**

```
ATGTATTTAGGAAACTATTTTGCATCTTTAAACAATTGAAGG|TCATTAAAT|AAAACGTGCATATTTTTGTGAGGTGTGAAATGTTTG
 >chr3:191669640-191669682 + E=1e-13                >chr3:191668672-191668791 + E=7e-60
AAAGACTGAATATTTATCCAAAAGAGATTGAGTCTTGCAACCCTAATAAAATAACTTATTCAAGCAAAGATCAACAGTGGACA|ATGAA
 p=4e-03
TTACTTTGGACATTCAAATGATTCAGTTAAACTAGGTATGTTAACAATTAATTAATTGAATTAACAAAACTGTTAATTCACTTGCTAAT

GTA|CTCAGTTTCCAGAACAATGCCTGGGGCACAGAGAAGGAGCTCAGTAAATATTTTAGTAAGTAAATGAATGAACAATCATTTGGGA
    >chr3:188573220-188573462 - E=6e-134
GTACTCCAAAGATTCATCCAACAGCATCTACTCCCATAGACATCCCCCAAATTTCATTACTATCCTCAGATGGCCTGCATCACCACACT

ATTTCAGCAGCCCCCTCTCCAGAGCCAGACTCTGTCTCTTTATCCTGTTGCTTTCTTCATAGTGCTTA|CTTGATATTTTTTATATCAA

AAATATTTTTCATCACTTAGTATAGAAATACTTAAAATATTATATATATTTTCTGTTATATAAATATAAT
```
